# Metabolic Profiling from an Asymptomatic Ferret Model of SARS-CoV-2 Infection

**DOI:** 10.3390/metabo11050327

**Published:** 2021-05-19

**Authors:** David J. Beale, Rohan Shah, Avinash V. Karpe, Katie E. Hillyer, Alexander J. McAuley, Gough G. Au, Glenn A. Marsh, Seshadri S. Vasan

**Affiliations:** 1 Land & Water, Commonwealth Scientific and Industrial Research Organisation, Dutton Park, QLD 4102, Australia or rshah@swin.edu.au (R.S.); avinash.karpe@csiro.au (A.V.K.); katie.hillyer@csiro.au (K.E.H.); 2 Department of Chemistry and Biotechnology, Faculty of Science, Engineering and Technology, Swinburne University of Technology, Hawthorn, VIC 3122, Australia; 3 Australian Centre for Disease Preparedness (ACDP), Commonwealth Scientific and Industrial Research Organisation, Geelong, VIC 3220, Australia; alex.mcauley@csiro.au (A.J.M.); gough.au@csiro.au (G.G.A.); glenn.marsh@csiro.au (G.A.M.); vasan.vasan@csiro.au (S.S.V.); 4 Department of Health Sciences, University of York, York YO10 5DD, UK

**Keywords:** animal model, COVID-19, ferret, lipidomics, metabolomics, SARS-CoV-2, systems biology

## Abstract

Coronavirus disease (COVID-19) is a contagious respiratory disease that is causing significant global morbidity and mortality. Understanding the impact of the Severe Acute Respiratory Syndrome Coronavirus-2 (SARS-CoV-2) infection on the host metabolism is still in its infancy but of great importance. Herein, we investigated the metabolic response during viral shedding and post-shedding in an asymptomatic SARS-CoV-2 ferret model (n = 6) challenged with two SARS-CoV-2 isolates. Virological and metabolic analyses were performed on (minimally invasive) collected oral swabs, rectal swabs, and nasal washes. Fragments of SARS-CoV-2 RNA were only found in the nasal wash samples in four of the six ferrets, and in the samples collected 3 to 9 days post-infection (referred to as viral shedding). Central carbon metabolism metabolites were analyzed during viral shedding and post-shedding periods using a dynamic Multiple Reaction Monitoring (dMRM) database and method. Subsequent untargeted metabolomics and lipidomics of the same samples were performed using a Liquid Chromatography Quadrupole Time-of-Flight Mass Spectrometry (LC-QToF-MS) methodology, building upon the identified differentiated central carbon metabolism metabolites. Multivariate analysis of the acquired data identified 29 significant metabolites and three lipids that were subjected to pathway enrichment and impact analysis. The presence of viral shedding coincided with the challenge dose administered and significant changes in the citric acid cycle, purine metabolism, and pentose phosphate pathways, amongst others, in the host nasal wash samples. An elevated immune response in the host was also observed between the two isolates studied. These results support other metabolomic-based findings in clinical observational studies and indicate the utility of metabolomics applied to ferrets for further COVID-19 research that advances early diagnosis of asymptomatic and mild clinical COVID-19 infections, in addition to assessing the effectiveness of new or repurposed drug therapies.

## 1. Introduction

The novel coronavirus disease, COVID-19, is currently one of the most significant health issues globally and the causative virus SARS-CoV-2 is still evolving, more than 15 months since the first reported case in Wuhan, China. Although the mortality rate is relatively low (2.1%), more than 3.25 million deaths have been reported worldwide as of 6 May 2021 [1]. Furthermore, the world is bracing for a new wave of infections from more contagious and pathogenic SARS-CoV-2 variants arising from the Brazil, India, South Africa, UK and the USA [2].

As the causative virus spreads through the respiratory system, the quickest and most employed method of diagnostics is based on polymerase chain reaction (PCR) testing of nasal and throat swabs, and less commonly used, anal swabs [3,4]. However, some of the predominant limitations of PCR methods, especially during the early stages of infection (<1 week), include the time required for a positive confirmation (up to 72 h) and the high technological inputs that are needed to perform the assay itself (i.e., PCR machines, primers, molecular probes among others) [5]. Other commonly used methods rely on the analysis of serology antibodies, and although cheaper and technically less challenging than PCR, they do not provide reliable results until the second week of infection, when the IgG and IgM antibodies peak in serum samples [5,6]. The biggest hurdle for both assays is the monetary price associated with each test. Although some countries include these tests in nationalized healthcare systems, the majority are expensive and can cost up to USD 486 per assay [7,8]. Furthermore, people subjected to a COVID-19 assay are asked to isolate or quarantine for a defined period until cleared, which is associated with an opportunity cost. This will increasingly become problematic if more regular testing is needed to ensure international borders remain open, that are free from enforced 14-day mandated hotel quarantine [9]. As such, a deeper understanding of COVID-19 infections, and disease progression through to recovery is needed to ensure a new COVID-normal society can be reached [10]; one that can capture asymptomatic and mild clinical infections and limit community transmission events.

One avenue to better understand the progression of this novel disease, and to inform health management decisions that potentially guide isolation/quarantine protocols, is through a deeper analysis of COVID-19 infections using metabolomics [11]. Metabolomics has been rapidly advancing clinical health research and can detect the subtle biochemical changes caused by disease pathogenesis [12,13,14,15]. The wide biochemical screening and high-throughput nature of metabolomics can enable a deep biochemical analysis of a disease state to occur within hours rather than days [16]. As the central metabolic pathways are generally conserved during evolution, this approach enables better comparison and correlation between ex vivo models, in vivo models, and human clinical data. Harnessing these metabolic outputs can potentially lead to the discovery of signature metabolic biomarkers relevant to pathogenesis that can be utilized for designing Point-of-Care (PoC) testing regimens for more rapid testing [17].

Evidence from the literature suggests this potential could soon become a reality. For example, Bruzzone et al. [18] performed NMR-based metabolomics and lipidomics on serum samples from 398 COVID-19 acute phase patients. It was reported that high levels of ketone bodies (acetoacetic acid, 3-hydroxybutyric acid, and acetone) were used as an alternative source of energy in COVID-19 patients [18]. The elevated levels of succinic acid and pyruvic acid were correlated with impaired central metabolism and/or mitochondrial metabolism. The presence of hepatic oxidative stress biomarker, 2-hydroxybutyric acid, revealed general metabolic stress in COVID-19 patients [18]. Shen et al. [19] performed proteomic and metabolomic profiling of sera and detected dysregulated metabolites involved in lipid metabolism. It was reported that the accumulation of steroid hormones including progesterone, androgens, and estrogens in COVID-19 contributed to macrophage modulation. Overmyer et al. [20] identified lipid transport dysregulation and sets of covarying molecules that are strongly associated with the status and severity of COVID-19. Wu et al. [21] found that the development of COVID-19 resulted in altered energy metabolism and hepatic dysfunction. In the case of hepatic dysregulation, carbamoyl phosphate of the urea cycle was the affected metabolite and was severely downregulated in fatal cases [21]. Here, Wu et al. stated that a significant difference in guanosine monophosphate was observed between healthy subjects and COVID-19 patients and that levels were also significantly different between mild and fatal cases [21].

While these metabolomics studies to date have all been reported from human observational studies involving infected COVID-19 patients, the variance in human populations, multiple contributing (health-related) co-factors, and the lack of control on external environmental conditions, means that more work is still needed to better understand and characterize these changes at the biochemical level in humans. An alternative approach is to investigate COVD-19 using an animal model in the first instance [22], exploring the infection interactome and characteristics at a level of ‘controlled’ detail and complexity not possible in an observational study that involves people, with the aspirational goal to translate those animal model findings into human clinical trials for validation and benchmarking. A similar approach has been taken in the development and efficacy assessment of novel vaccine and drug therapeutics for COVID-19 [23] and influenza [12]. As such, here we aimed to explore COVID-19 using an established (asymptomatic/mild infection) SARS-CoV-2 ferret model [24,25], specifically looking at the changes in the metabolome during- and post-shedding from a range of commonly collected (and minimally-invasive) sample types; i.e., those samples typically collected when diagnosing humans for COVID-19, specifically: nasal washes, oral swabs, and rectal swabs. This study also included an assessment of two locally acquired SARS-CoV-2 isolates that were administered at different challenge dosages to simulate an asymptomatic/mild infection. Through the utility of an established asymptomatic/mild infection animal model, we aim to demonstrate the value of metabolomics for informing better clinical diagnosis and facilitating therapeutic development via non-invasive sampling.

## 2. Results and Discussion

### 2.1. Determination of Virus Shedding

**Figure 1:**
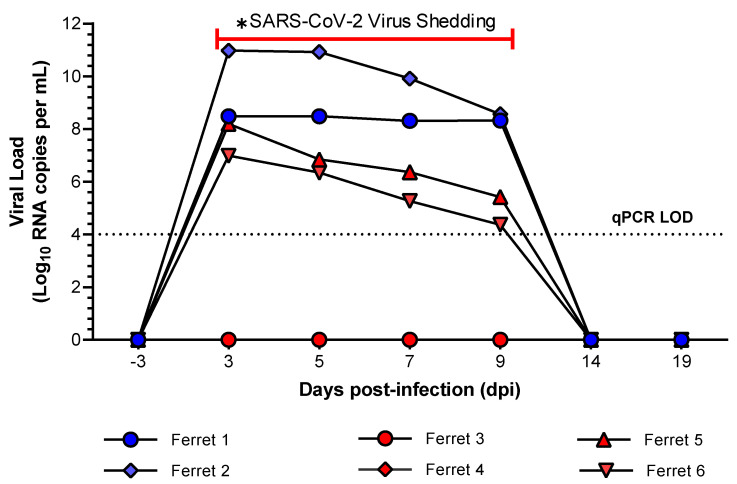
Determination of virus shedding in nasal washes collected from six ferrets challenged with SA01 (betaCoV/Australia/SA01/2020) and VIC01 (betaCoV/Australia/VIC01/2020) SARS-CoV-2 isolates. Ferrets 1 and 2 were challenged with 1 × 10^3^ TCID_50_ SARS-CoV-2 VIC01; ferrets 3 and 4 were challenged with 5 × 10^2^ TCID_50_ SARS-CoV-2 SA01; ferret 5 and 6 were challenged with 6 × 10^5^ TCID_50_ SARS-CoV-2 SA01. Nasal wash samples from SARS-CoV-2 VIC01 infected ferrets are annotated blue and SARS-CoV-2 SA01 infected ferrets are annotated red. SARS-CoV-2 RNA was detected in nasal wash samples from ferrets 1, 2, 5, and 6 on 3, 5, 7, and 9-days post-infection (dpi). The dotted line box represents the limit of detection (LOD) of the reverse transcription qPCR assay. Part of this figure contains published data found in Marsh et al. [25]. The asterisks represents the defined period for SARS-CoV-2 virus shedding used to define sample groupings.

Virus shedding from ferrets challenged with two locally acquired SARS-CoV-2 isolates, betaCoV/Australia/SA01/2020 (SA01) and betaCoV/Australia/VIC01/2020 (VIC01), was determined from nasal washes and, oral and rectal swabs by reverse transcription quantitative-PCR (qPCR). SARS-CoV-2 RNA was detectable in the nasal washes collected from four of the six ferrets on 3, 5, 7, and 9-days post-infection (dpi) (Figure 1). Viral RNA was only detected in one oral swab sample (9 dpi) from one of the ferrets (data not shown); no virus RNA was detected in the rectal swabs in any ferret, at any time point. While the dose of the viral challenge differed between the two isolates, the 1 × 10^3^ TCID_50_ VIC01 and 6 × 10^5^ TCID_50_ SA01 doses were deemed comparable and sufficient in providing positive viral replication in the nasal wash samples in Ferret 1, 2, 5, and 6 (Figure 1). This variation of infection dose between the two isolates was needed to ensure a qPCR positive infection response was observed in the ferrets when challenged (as determined by the viral shedding data; Figure 1). A lower challenge dose of the SA01 isolate (5 × 10^2^ TCID_50_ SARS-CoV-2 SA01), which was considered comparable to the VIC01 dose, did not provide a positive qPCR result in any of the samples collected and these SA01-challenged ferrets (Ferrets 3 and 4) were not used in the subsequent metabolomics analysis described below. It has been hypothesized that “the dose of virus in the initial inoculum” is linked to patient COVID-19 severity [26], and that the inoculum dose of variants and sub-types can alter this inoculum viral dose further [27]. Interestingly, once infected, asymptomatic patients have similar viral shedding profiles [28]. This is evident in the viral load data of the ferret model used in this study. The challenge dose between the two isolates was altered to obtain a positive asymptomatic response which was confirmed by qPCR. Once positive, the viral infection load was comparable irrespective of the original inoculum dose. The utility of the qPCR negative samples as a control was not considered in the statistical analysis. This is due to the samples numbers within this group being considered too small for a meaningful statistical analysis and the fact that these ferrets were challenged, albeit unsuccessfully, they may be metabolically altered because of the failed challenge and not representative of a true negative control.

These results indicate that nasal wash samples are the most appropriate sample type to detect changes in the biomolecules in ferrets resulting from a COVID-19 infection. Again, with the ferret model herein being representative of an asymptomatic/mild infection, the utility of nasal wash samples may pave the way forward for profiling and understanding the disease’s progression in similarly mild or asymptomatic human patients using a biofluid that is relatively easy to collect and minimally invasive to patients [29].

No significant differences in terms of viral shedding and animal physiology were observed for the two isolates studied. Temperature and weight measurements were collected daily, and values remained within normal limits (see Marsh et al. for these data [25]). Based on these results, and accounting for the limitation of the animal ethics permit granting the utility of only six ferrets in this instance, the samples collected on 3, 5, 7, and 9 dpi from the four infected ferrets were grouped as ‘shedding’ samples; and samples collected on 14, 19, and 25 dpi were grouped as ‘post-shedding’ samples. Whilst the infected ferrets did not develop COVID-19 symptoms typically observed in humans, they do support viral infections with quantifiable outputs that make them appropriate for further evaluation as a model of mild or asymptomatic human disease using metabolomics-based approaches [25].

### 2.2. Central Carbon Metabolism Variance in Collected Biological Sample Types

**Figure 2:**
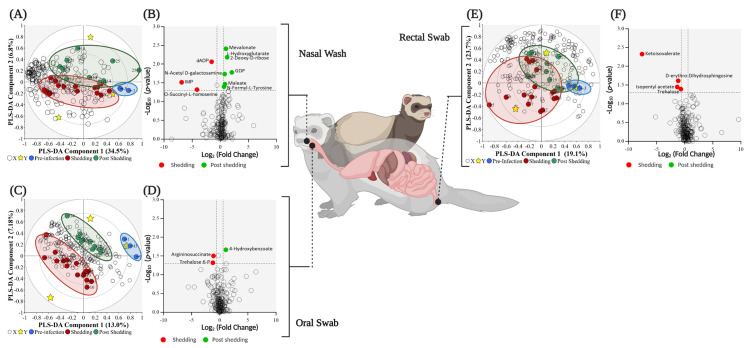
Biplot overview (**A**,**C**,**E**) of central carbon metabolism metabolites and associated volcano plots (**B**,**D**,**F**) of the significant metabolites using a fold change threshold of ≥1.5 or ≤0.66 and a *p*-value of ≤0.05. (**A**,**B**) nasal washes (R^2^X = 0.414, R^2^Y = 0.502, and Q^2^ = 0.175); (**C**,**D**) oral swabs (R^2^X = 0.428, R^2^Y = 0.336, and Q^2^ = −0.134), and (**E**,**F**) rectal swabs (R^2^X = 0.202, R^2^Y = 0.628, and Q^2^ = −0.148). The ellipse on the biplots represent the 100%, 75% and 50% correlation coefficient for measured metabolites. For panels (**A**,**C**,**E**), the colored circles represent each analyzed sample, while the yellow-colored stars indicating the averaged group position for each sample cluster, and the white circles representing the distribution of central carbon metabolism metabolites between these groups. In the volcano plots (panels **B**,**D**,**F**), the red circles represent significant metabolites identified during shedding, the green circles represent significant metabolites post-shedding, and the white circles represent the non-significant metabolites.

Firstly, the infected ferret samples were subjected to a central carbon metabolism metabolite screening via a Liquid Chromatography Triple Quadrupole Mass Spectrometry (LC-QqQ-MS) method. The nasal washes, as well as the oral and rectal swabs, indicated the presence of 139 out of the 223 common polar compounds from the central carbon metabolism metabolites and related pathways. This metabolomics dataset was processed via an unsupervised statistical approach using Principal Component Analysis (PCA). Any sub-data clustering was not evident from the PCA analysis (Supplementary Figure S1). Most of the samples were fitted within the DCrit threshold of the distance of observation (DModX) analysis, indicating them to be non-outliers (Supplementary Figure S1). The grouped data, for the different non-invasive sample types (nasal washes, oral and rectal swabs), were then analyzed using a supervised partial least squares discriminant analysis (PLS-DA) to explore differences during viral shedding and non-shedding time points (Figure 2A,C,E). Ideally, analysis of the individual sampled time points would be more desirable here, however, due to the restricted number of animal replicates allowed by the animal ethics committee approving this work, it was necessary to pool sample data within these well-defined groups and restrict the interpretations to explore viral shedding and post-shedding events. Consideration of the pre-challenge samples (−3 dpi) was determined not statistically significant. Overall, the PLS-DA analysis of nasal wash samples resulted in better separation (Q^2^ = 17.5%, Figure 2A) of the grouped data when compared against rectal swabs (Q^2^ = −14.8%, Figure 2C) and oral swabs (Q^2^ = −13.4%, Figure 2E). Furthermore, the nasal wash PLS-DA dataset was the only model that proved marginally statistically significant (*p*-value = 0.103) when cross-validated.

Fold change (FC) analysis of the 139 central carbon metabolism metabolites revealed that the abundance of 68, 104, and 91 metabolites increased post-shedding in the nasal wash, oral swabs, and rectal swabs, respectively (FC > 1.5). However, not all these features were found to be statistically significant (*p*-value ≤ 0.05). The metabolic variations between grouped data in each sample are shown in associated volcano plots in Figure 2. The volcano plot shows a total of 17 statistically significant metabolites—the nasal washes contained 3 and 7 elevated metabolites in shedding and post-shedding groups, respectively (Figure 2B); oral swabs contained 2 and 1 elevated metabolite in shedding and post-shedding groups, respectively (Figure 2D); and rectal swabs contained 4 elevated metabolites in the shedding group only (Figure 2F). The number of metabolites, whose abundance significantly altered due to viral infection, was higher in the nasal washes than oral swabs or rectal swabs. The reduced number of significant metabolites in the latter (i.e., oral swabs and rectal swabs) may be an artifact of the ferrets being asymptomatic (i.e., no clinical signs of SARS-CoV-2 infection) and the majority of the viral replication occurring in the nasal wash samples, as confirmed by qPCR (Figure 1). These results further support the hypothesis that nasal washes are better suited for investigating the biochemistry of respiratory infections during and post-viral shedding.

### 2.3. Chemical and Pathway Enrichment Analysis

**Figure 3:**
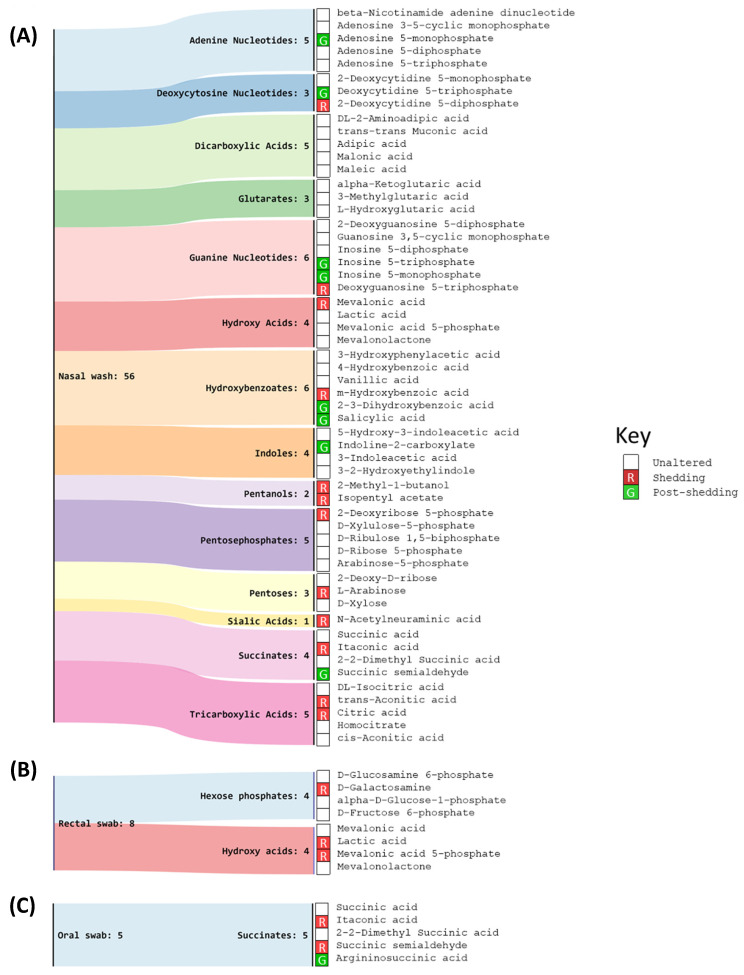
Identified metabolite clusters and the associated significant metabolites during viral shedding (green) and post-shedding (red) based on the acquired targeted central carbon metabolism metabolite dataset using the ChemRICH analysis tool. (**A**) nasal washes, (**B**) oral swabs, and (**C**) rectal swab. Altered significant metabolites (FC ≥ 1.5 or FC ≤ 0.66; *p*-value ≤ 0.05) identified in each cluster are illustrated in the heatmaps. The significant metabolites during viral shedding event are represented in red and those during post-shedding are represented in green in the heatmaps. The unaltered metabolites are represented in white.

We performed a chemical similarity enrichment analysis using ChemRICH [30] on the central carbon metabolism dataset. This provided chemical class-based information of significantly altered metabolites in each sample type analyzed. ChemRICH identifies highly impacted compound classes through the generation of metabolite clusters based on chemical similarity and ontologies that are not defined by organism-specific metabolic pathways which can be inherently flawed [30]. ChemRICH analysis does not rely upon background databases for statistical calculations. The most impacted compound clusters are summarized in Figure 3 and further provided in Supplementary Table S1.

**Figure 4:**
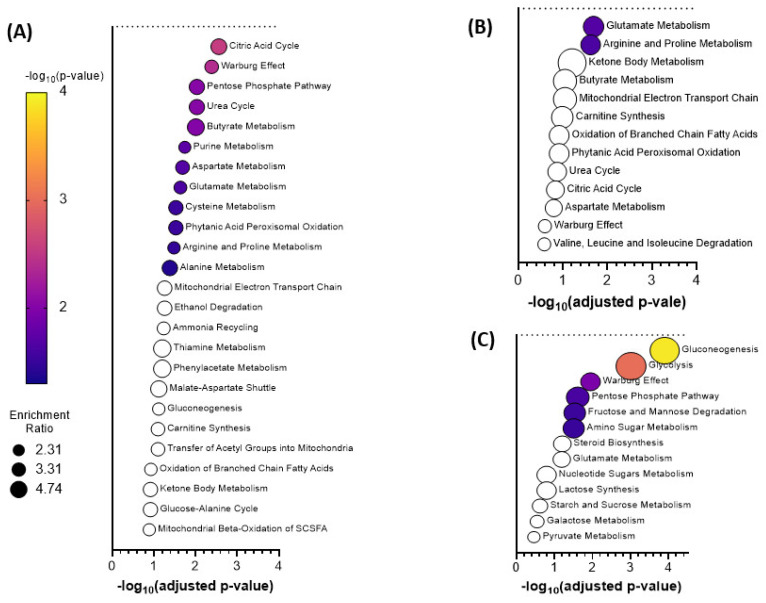
Significant pathways identified via the enrichment analysis of the identified significant metabolites and their associated ChemRICH chemical clusters during shedding and post shedding events of the (**A**) nasal wash, (**B**) oral swabs, and (**C**) rectal swab samples using MetaboAnalyst 5.0 (Enrichment Analysis Toolbox). Pathways annotated with white circles were enriched but found not to be significant.

In the oral swab samples, succinates, and, in the rectal swab samples, hexose phosphates and hydroxy acids, were significantly altered. Several more chemical classes were altered in the nasal wash samples including indoles, adenine nucleotides, succinates, deoxycytosine nucleotides, dicarboxylic acids, glutarates, guanine nucleotides, hydroxy acids, hydroxybenzoates, pentoses, pentanols, pentose phosphates, sialic acids, and tricarboxylic acids. The metabolites from these chemical clusters were used for pathway enrichment analysis (Figure 4). We further subjected these data from each sample type for enrichment analysis based on the Small Molecule Pathway Database (SMPDB) [31].

Pathway analysis of the identified significant metabolites and their chemical cluster metabolite partners, from the shedding versus post-shedding groups, that were detected in nasal wash samples. The pathway analysis indicated a significant (*p*-value ≤ 0.05) change in the citric acid cycle, purine metabolism, and pentose phosphate pathway. Out of 10 significant metabolites identified earlier, five are from the significant metabolite clusters identified in the ChemRICH analysis (L-hydroxyglutaric acid, mevalonate, 2-deoxy-D-ribose, inosine-5-monophosphate, and maleic acid). Several other important metabolic pathways including the Warburg effect, urea cycle, amino acid metabolism (aspartate, alanine, cysteine, glutamate, arginine, and proline), thiamine metabolism, phytanic acid metabolism, butyrate metabolism, mitochondrial electron transport chain and ammonia recycling were also identified as being significantly altered (1.5 ≥ FC ≤ 0.66; *p*-value ≤ 0.05) in nasal wash samples, as indicated by the enrichment analysis (Figure 4A). Metabolites associated with glycolysis, amino sugar metabolism, pentose phosphate pathway, fructose and mannose degradation, and gluconeogenesis were significantly altered (*p*-value ≤ 0.05) in rectal swab samples (Figure 4B). None of these metabolites were represented in the ChemRICH analysis of the rectal swabs. The glutamate metabolism and arginine and proline metabolism were the most significantly perturbed pathways in oral swab samples (Figure 4C).

### 2.4. Untargeted Metabolomics and Lipidomics of Nasal Wash Samples

As the nasal wash samples proved most informative based on the qPCR viral shedding and central carbon metabolism metabolite data, further analysis of these samples was then performed using an untargeted metabolite and lipid analysis using a Liquid Chromatography Quadrupole Time-of-Flight Mass Spectrometry (LC-QToF-MS) method. The untargeted analysis of the nasal wash samples indicated the presence of 2427 polar metabolites features. Of these, 341 polar metabolites were identified using a PCDL database generated from the central carbon metabolism metabolite outputs, known metabolite literature identified in human observational studies, and the commercial METLIN database [32].

**Figure 5:**
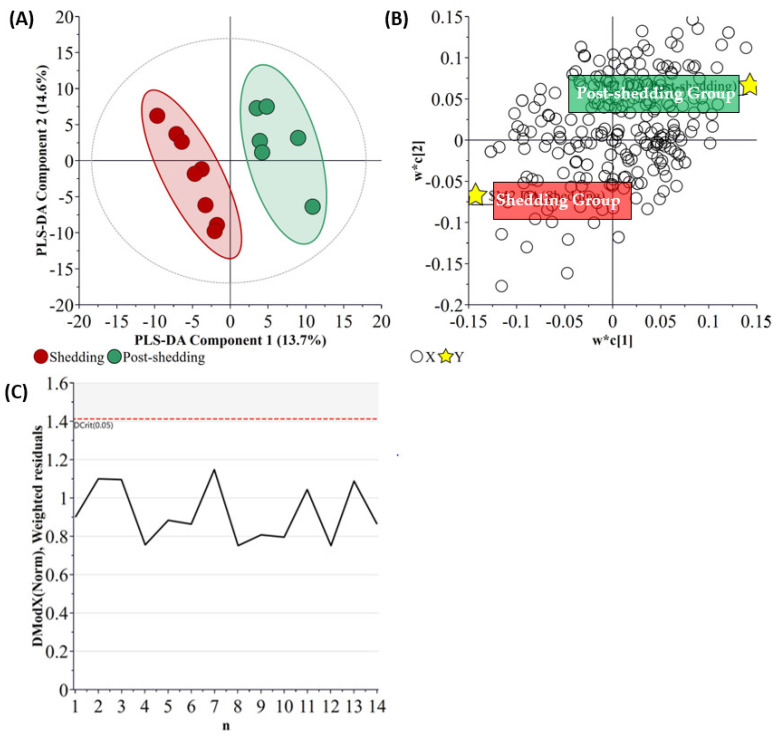
Partial least square-discriminant analysis (**A**) scatter plot, (**B**) loading plot, and (**C**) distance of observation plot of nasal wash samples collected from Ferrets. R^2^X (cum) = 0.535, R^2^Y (cum) = 0.999, Q^2^ = 0.650. The ellipse presented in Figure 5A represents Hotelling’s T2 confidence limit (95%). The colored circles in panel (**A**) represent each analyzed sample, while the yellow-colored stars in panel (**B**) indicate the average group position for each sample cluster, with the white circles representing the distribution of metabolite features between these groups.

The PLS-DA analysis of the untargeted acquired data from the nasal wash samples (Figure 5) resulted in better model separation (Q^2^ = 65%) compared to the model created using the central carbon metabolism metabolite acquired data (Q^2^ = 17.5%). It is noted that all these samples were fitted within the DCrit threshold of the distance of observation (DModX) analysis, indicating them to be non-outliers. Cross-validation of this model resulted in a *p*-value of 0.031.

Twelve polar metabolites were identified as statistically significant by FC analysis; eight of them downregulated and four upregulated in the post-shedding group. The downregulated metabolites are L-1-pyrroline-3-hydroxy-5-carboxylate, pyroglutamic acid, (R)-(+)-2-pyrrolidone-5-carboxylic acid, leukotriene A_4_, choline, butyric acid, niacinamide, and 2-amino-tetradecanoic acid. The upregulated metabolites are allantoic acid, 2-aminobut-2-enoate, 2-iminobutanoate, and indole acetaldehyde. The untargeted LC-QToF-MS lipidomic analysis of the nasal samples indicated only three statistically significant lipids out of a total of 325 identified lipids. The statistically significant lipids including several phosphatidylethanolamine class of lipids (PE), namely, PE(20:4/22:6), PE(20:5/22:5), and PE(22:6/20:4) which were all downregulated in the post-shedding group.

**Figure 6:**
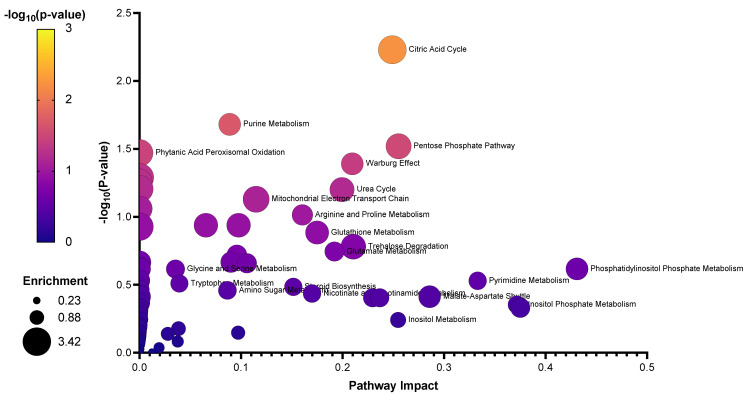
Enrichment analysis of the identified metabolites and lipids of importance from nasal wash samples.

**Figure 7:**
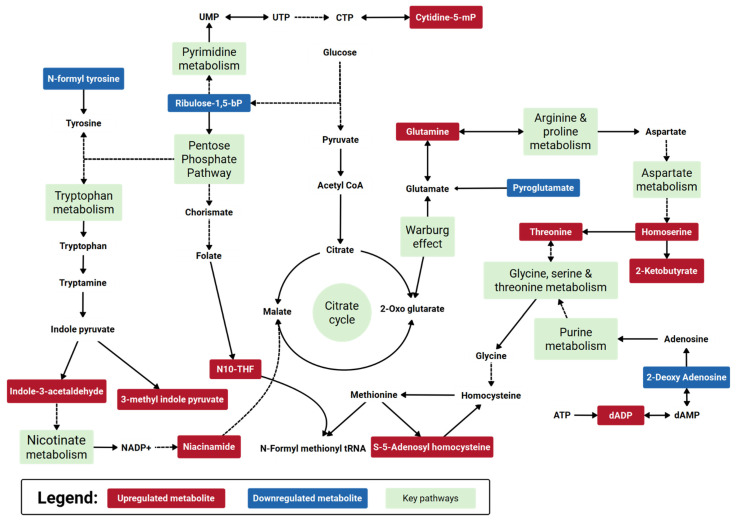
Curated metabolic pathway of key metabolism changes in a SARS-CoV-2 ferret model during post- shedding. Note, red-colored metabolites are identified features that are upregulated during post-shedding relative to viral shedding events; blue-colored metabolites are identified features that are downregulated during post-shedding relative to viral shedding events. Key metabolic pathways of interest identified from the pathway enrichment and impact analysis are annotated green. The data for individual pathway metabolites is presented in the Supplementary Figure S3.

The key compounds identified in the central carbon metabolism LC-QqQ-MS metabolomic analysis, untargeted LC-QToF-MS metabolomic analysis, and untargeted LC-QToF-MS lipidomic analysis were subsequently used for metabolite and lipid enrichment analysis using MetaboAnalyst 5.0 (Figure 6 illustrated a combined graphical overview). Supplementary Figure S2A,B provide the enrichment and pathway impact analysis of the individual metabolite datasets, while Supplementary Figure S2C,D provide the enrichment analysis and pathway impact analysis of the individual lipid dataset. As a means of synthesizing these findings further, a curated metabolic pathway of the significant and important metabolites and pathways was generated (Figure 7), to which future research efforts using SARS-CoV-2 animal models can be validated and benchmarked against.

Pathway analysis between shedding and post-shedding groups of the nasal washes collected from infected ferrets showed several perturbed pathways (predominantly amino acid-related). Among the key metabolites identified; we found argininosuccinic acid and L-1-pyrroline-3-hydroxy-5-carboxylate involved in the arginine and proline metabolism and succinic semialdehyde involved in glutamate metabolism. Arginine and proline metabolism is related to clinical evolution and COVID-19 disease progression, with arginine an essential amino acid for nitric oxide homeostasis [33]. Our findings are consistent with the observations of Blasco et al. [33] and Shen et al. [19] regarding the enrichment of metabolites involved in arginine metabolism.

Perturbation in energy metabolisms such as citric acid cycle (TCA cycle), pentose phosphate pathway, and urea cycle was also observed. Several metabolites of the citrate cycle were slightly elevated (FC ca. 1.10) post shedding but not considered statistically significant (*p*-value > 0.10); these included cis-aconitic acid, citric acid, isocitric acid, α-ketoglutaric acid, succinic acid, adenosine 5-triphosphate, β-nicotinamide adenine dinucleotide and adenosine 5-diphosphate. Downregulation of the citrate cycle during virus shedding could be due to the high energy consumption caused by the virus [21]. For example, COVID-19 health care practitioners account for energy expenditure increases of up to 10% during asymptomatic viral infections [34]. Such a decrease in the citrate cycle metabolism would cause an imbalance of the anti-oxidization mechanism and inflammatory damage [35]. During the shedding period, the downregulation of citrate correlated with a significant increase in glutamate and arginine metabolisms. It is a known phenomenon that during infections, the viral systems tend to hijack the host amino acid metabolism, especially the glutamate metabolism [36], leading to glutaminolysis, to increase the pathogenesis. Genomic studies on *Pasteurella multocida* infections indicated that glutamine promoted the expression of parasitic virulence factors in the lungs [37]. Conversely, macrophages are also known to prefer glutaminolysis during parasitation [38]. The observations here were also found in our previous H1N1 infection study that showed elevated levels of glutamate at 1–3 dpi and downstream metabolites such as aspartyl-glutamate and allothreonine at about 4–6 dpi [12] in nasal washes collected from ferrets challenged with influenza.

Tryptophan degradation was also predominant in the nasal washes during the shedding period. Recently reported studies have shown that during respiratory syncytial virus (RSV) infection in BALB/c mice, elevated indole levels and depleted 5-hydroxy indole acetate were observed in the lungs [39,40]. Our study showed that SARS-CoV-2 infection caused a slightly altered tryptophan metabolism where the predominant metabolites elevated were indole acetaldehyde and 3-methylindole pyruvate (Supplementary Figure S3). However, it should be noted that although both SARS-CoV-2 and RSV target the respiratory system, SARS-CoV-2 affects the upper respiratory tract [41] more than the lower respiratory system, which is the case with RSV [39]. This difference possibly caused the diversion of tryptophan metabolism to indole pyruvate and indole acetate production, rather than the production of stress metabolites such as l-kynurenine, 5-hydroxy indole acetate, and serotonin. Lin et al. [42] studied the effects of viral pneumonia on different mouse organs. In their study, the perturbation of tryptophan metabolism was observed in serum samples only and not in the respiratory system. In this study, it is most likely that these metabolites are captured in the nasal washes via the blood-gas interphase in the lungs, indicating a greater potential of nasal washes to being a superior minimally invasive sample type for rapid identification or characterization of such infections.

Metabolites of the purine and pyrimidine pathways such as xanthine, hypoxanthine, and thymine were also identified in the challenged ferrets. These metabolites may be linked with purine and pyrimidine release from cell lysis [43]. Purines such as these play an important role in regulating the activation and differentiation of immune cells. Environmental factors such as hypoxia can modulate the release of purines and pyrimidines, in turn, can control the inflammation induced by virus infection [33]. In the current study, purine metabolism intermediates such as 2-deoxyadenosine-5-diphosphate were elevated in the nasal washes during the shedding period, which correlated to the enriched thiamine metabolism pathway downstream (Figure 7). In vitro, culture experiments show that a combination of riboflavin (vitamin B2) and UV light decreases the SARS-CoV-2 titers [44]. Similarly, thiamine supplementation in patients has been shown to lower the IL-17 pro-inflammatory immune storm and simultaneously increase the anti-inflammatory IL-22 levels [45]. Other impacted pathways that we observed are also common in clinical observational studies of COVID-19 infected patients. For example, the Warburg effect has been identified as having a key role in SARS-CoV-2 replication and associated inflammatory response [46]. Others have demonstrated the downregulation of the pentose phosphate pathway [47]. While others have reported the role of nicotinate metabolism linked to inflammatory signals [33].

### 2.5. Specificity of SARS-CoV-2 Virus Isolates

**Figure 8:**
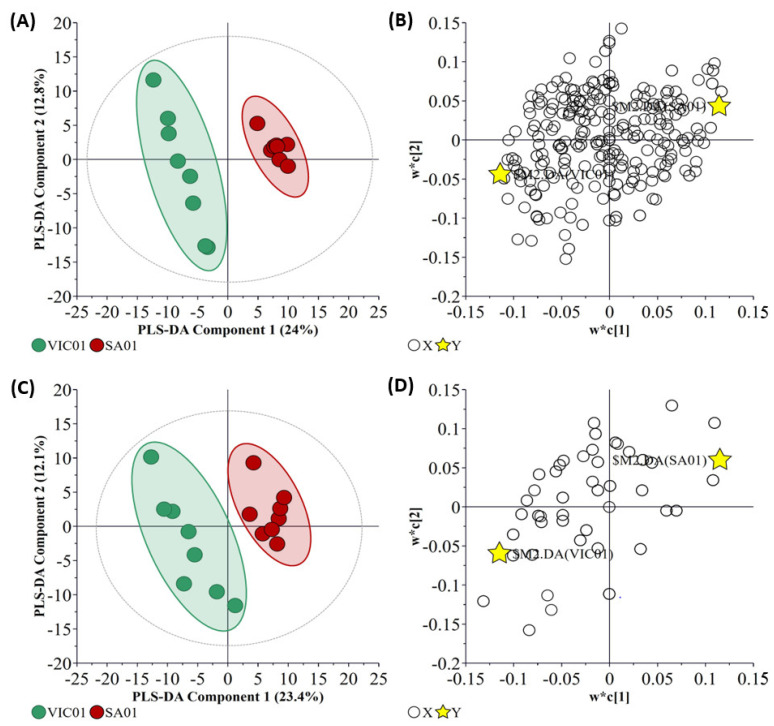
Overview of the LC-QToF-MS untargeted metabolite (R_2_*X* = 0.367, R_2_Y = 0.989, and Q_2_ = 0.872, DCrit value = 1.36181) and lipid (R_2_*X* = 0.356, R_2_Y = 0.935, and Q_2_ = 0.656, DCrit value = 1.34667) data from the two inoculated virus variants analyzed in nasal wash samples during viral shedding. (**A**) PLS-DA score scatter plot of metabolite data. (**B**) PLS-DA loading scatter plot of metabolite data. (**C**) PLS-DA score scatter plot of lipid data. (**D**) PLS-DA loading scatter plot of lipid data. The ellipse on the loadings scatter plots represents the 95% Hotelling’s threshold. Note, the ellipse presented in Figure 8A,C represents Hotelling’s T2 confidence limit (95%). Note: The colored circles in panels (**A**,**C**) represent each analyzed sample, while the yellow-colored stars in panels (**B**,**D**) indicate the average group position for each sample cluster, with the white circles representing the distribution of metabolite/lipid features between these groups.

The polar metabolites and lipids from the nasal wash samples of ferrets challenged with the two SARS-CoV-2 isolates were analyzed to identify isolate-dominated metabolites/lipids and related pathways. This is particularly important when assessing the metabolic response of SARS-CoV-2 variants and investigating their pathogenicity. It is noted that the clinical presentation of these isolates in humans is considered equivalent (mild-moderate), with the key point of difference being the virus inoculum dose needed to cause an infection in ferrets. The PLS-DA analysis of metabolite and lipid data is illustrated in Figure 8. The metabolite data (Q^2^ = 0.872) analysis showed a better separation than lipid data (Q^2^ = 66%). Cross-validation of these models was found to be statistically significant (*p*-value of 0.0203 and 0.0007, respectively).

**Figure 9:**
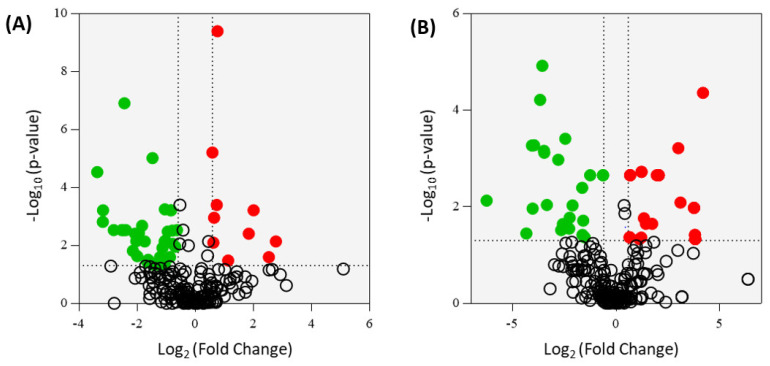
Volcano plots of the significant metabolites from central carbon metabolism (**A**) and untargeted metabolites (**B**) using a fold change (FC) threshold of ≥ 1.5 or ≤ 0.66 and a *p*-value of ≤0.05. For a full list of significant metabolites, see Supplementary Information, Tables S2 and S3. The red circles are the significant metabolites that were upregulated in the ferrets infected with the SA01 isolate relative to the VIC01 isolate. The green circles are the metabolites that were downregulated in the SAC01 isolate relative to the VIC01 isolate.

The FC analysis of the metabolites from the central carbon metabolism and untargeted metabolites is illustrated in Figure 9. A total of 89 metabolites were found to be statistically significant (Supplementary Tables S2 and S3). Twenty-seven metabolites were upregulated, while 62 metabolites were downregulated in nasal washes of ferrets infected with the SA01 isolate. Lipidomic analysis indicated a total of 77 significant (*p* ≤ 0.05) lipids, of which 10 were identified (Supplementary Table S4); for the SA01 isolate infected ferrets, five lipids were significantly elevated, and five lipids were significant elevated in the VIC01 isolate infected ferrets.

**Figure 10:**
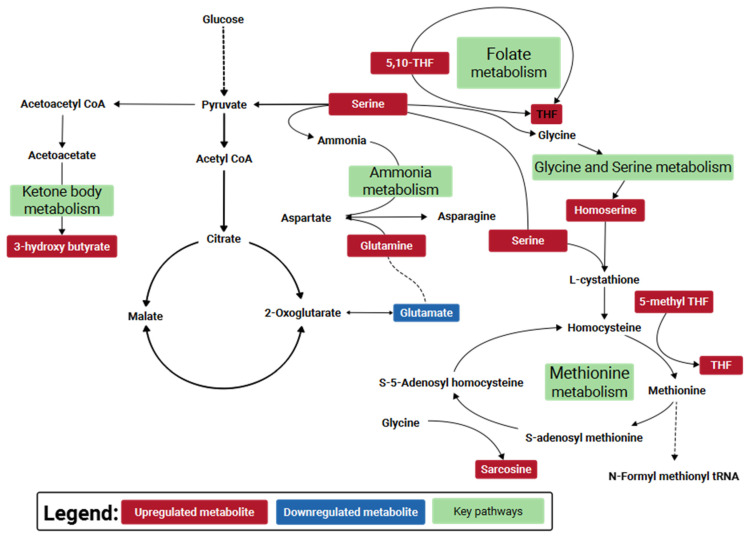
Curated metabolic pathway of key metabolism changes in a SARS-CoV-2 ferret model infected with the SA01 isolate during virus shedding. Note, red-colored metabolites are significant features that are upregulated during viral shedding when infected with SA01 isolate. Key metabolic pathways identified from the pathway enrichment and impact analysis are annotated green. The data for individual metabolites is presented in the Supplementary Figure S4.

A comparison of the host response to SA01 and VIC01 isolates showed that most of the above-mentioned metabolites, which resulted in a higher host response, were considerably elevated in the SA01 isolate challenged ferrets. Especially, the kynurenines, which were not statistically significant in the ‘shedding vs. post shedding’ groupings described above but were found to be significantly upregulated in the SA01 challenged ferret nasal washes here. Several other metabolic pathways were significantly (*p*-value < 0.1) enriched in the SA01 isolate (Figure 10) that resulted in the identification of additional enriched pathways that involved selenoamino acid metabolism (*p*-value < 0.1), folate metabolism (*p*-value < 0.1), urea cycle (*p*-value < 0.1), ketone body metabolism (*p*-value < 0.1), and glucose-alanine cycle (*p*-value < 0.1). On the other hand, no metabolic pathways were found to be significantly enriched in the VIC01 isolate infected ferrets.

These results indicate that the ferrets infected with the SA01 isolate were more affected, compared to the VIC01 isolate. Following other respiratory viral infection studies [39,40], the observations here point to a higher likelihood that, for the ferrets infected with the SA01 isolate, both the upper and lower respiratory tracts were perturbed compared to the VIC01 isolate. For example, a considerable elevation of nicotinamide adenine dinucleotide (NAD) and N10-THF indicates a significantly elevated host immune response to the SA01 isolate [39,40]. Additionally, the elevated NAD levels (Supplementary Figure S4) and upregulated Warburg effect, also indicated towards a likelihood of Acetyl CoA diversion pathway being active in the nasal environment during the infection [48], particularly during infection by SA01 variant. Furthermore, the PE lipids, which form part of a cell membrane and provide the cell signaling function to the phagocytes during cellular phagocytes [49], are differentially expressed. Recent research suggests that viral pathogens hijack these PE signaling systems to improve their replications. Particularly, the study by Barberis et al. [50] indicated that the PE lipids showed accumulation in blood samples of the patients infected with SARS-CoV-2. Our study here showed that the ferret model also expresses these lipids at elevated levels in the nasal washes of the SA01 isolate challenged ferrets. The previous studies have shown that during the SARS infection, the SARS-CoV fusion proteins cause host cell membrane destabilization by specifically causing the curvature stresses on the PE membranes [51]. The SARS-CoV-2 infection causes significant damage in the lungs, triggering an abnormal clotting cascade [52]. Therefore, a further, larger study, building on the output of the current study herein will be able to validate the critical role of PE lipids and other metabolites as a potential biomarker that could enable a rapid differentiation of various SARS-CoV-2 strains. Furthermore, histologic examination of biopsy tissues during SARS-CoV-2 shedding or autopsy tissues post shedding that characterizes damaged tissues hypothesized from the altered biochemistry observed in the metabolomics is needed to assess the biochemical implications of an asymptomatic infection.

While this study is limited in terms of the number of biological replicates, it should be noted that the utility of four infected asymptomatic ferrets during viral shedding and post shedding events does meet the minimum reporting requirements stipulated by the Metabolomics Standard Initiative (MSI). The MSI sets the minimum number of biological replicates to three [53]. The authors concede that the use of a limited number of samples has the potential to introduce self-association and statistical biases in the data. More so for the comparison between the two selected isolates used in this study that further reduce the biological replicates per isolate to two ferrets. This limitation is overcome by expanding the number of collections per biological replicate in each of the infected ferrets over the duration of the shedding and post-shedding events. As such, these data provide a proof-of-concept application that differentiates ferrets infected by different SARS CoV-2 isolates. Moreover, this preliminary study has demonstrated the value of metabolomics applied to an asymptomatic SARS-CoV-2 ferret model, and to this end, these data formed the basis for a follow up study to be approved that increases the biological replicates significantly (n = 20). Importantly, the metabolic profiling from an asymptomatic ferret model of SARS-CoV-2 infection herein clearly indicates the great potential of metabolomics for better understanding the biochemical change associated with the infection and the value metabolomics provides for informing better clinical diagnosis and facilitating therapeutic development via non-invasive sampling approaches.

More work is currently underway to characterize the asymptomatic SARS-CoV-2 ferret model, as alluded above, utilizing a variety of variants and mutations coupled with vaccine and therapeutic therapies available for human use over the duration of a COVID-19 infection and post recovery. The intent is also to establish a parallel metabolome profile of minimally-invasive samples from a symptomatic SARS-CoV-2 model (hamsters). Collectively, these animal models will then guide human clinical studies that lead to the development of non-invasive typing technologies of COVID-19 severity, and via an understanding of disease progression in the asymptomatic and symptomatic animal models, and better understanding of disease progression and recovery for infected patients, and broader protection from community transmission.

## 3. Materials and Methods

### 3.1. Animal Ethics

This study was reviewed and approved by the Animal Ethics Committee (AEC) at the CSIRO Australian Centre for Disease Preparedness (AEC approval number #1989).

### 3.2. Ferret Challenge and Sample Collection

Six male, outbred ferrets, approximately 4 months of age, were housed in appropriate caging under PC-4 conditions at the CSIRO Australian Centre for Disease Preparedness (formerly Australian Animal Health Laboratory). Animal housing, husbandry, and handling for sample collections were as previously described Pallister et al. [54]. Before the challenge, ferrets were implanted with a LifeChip Bio-Thermo transponder (Destron Fearing); subcutaneous temperature, rectal temperature and body weight were recorded.

Two ferrets (1 and 2) were challenged with 1 × 10^3^ TCID_50_ SARS-CoV-2 VIC01, while the other four ferrets were challenged with 5 × 10^2^ TCID_50_ SARS-CoV-2 SA01 (ferrets 3 and 4) and 6 × 10^5^ TCID_50_ SARS-CoV-2 SA01 (ferrets 5 and 6). The GenBank accession numbers for the VIC01 and SA01 isolates are MT007544 and MT745746, respectively. Supplementary Table S5 provides a summary of the variations between these two isolates, relative to Wuhan-Hu-1 (NC_045512). In infected humans, the two isolates were clinically classified ‘mild-moderate’ following the World Health Organization minimal common outcome measure (after Bauer et al. [55]). Ferrets were challenged via the intranasal route (0.5 mL total volume diluted in PBS). The inoculum was back-titrated by TCID_50_ assay on Vero E6 cells to confirm the administered dose.

Following the challenge, animals were assessed at least once per day (twice-daily for days 2–12 post-challenge) for the presence of clinical signs such as reduced-interaction score [56], fever (microchip temperature) and respiratory disease. On days 3, 5, 7, 9, 14 and 19, ferrets were anesthetized for collection of nasal wash, oral and rectal swab samples, as well as for the measurement of rectal temperature and body weight (data not shown). All samples were rapidly frozen, gamma-irradiated (50 kGy), and stored at −80 °C before extraction and analysis. Gamma irradiation was performed to inactivate the SARS-CoV-2 virus and enable the safe removal of samples from the PC-4 containment laboratory where the ferret challenge experiments were conducted. On day 25 post-challenge, ferrets were humanely killed. Reverse transcription qPCR was performed as per Marsh et al. [25]. 

### 3.3. Metabolite and Lipid Sample Extraction

**Figure 11:**
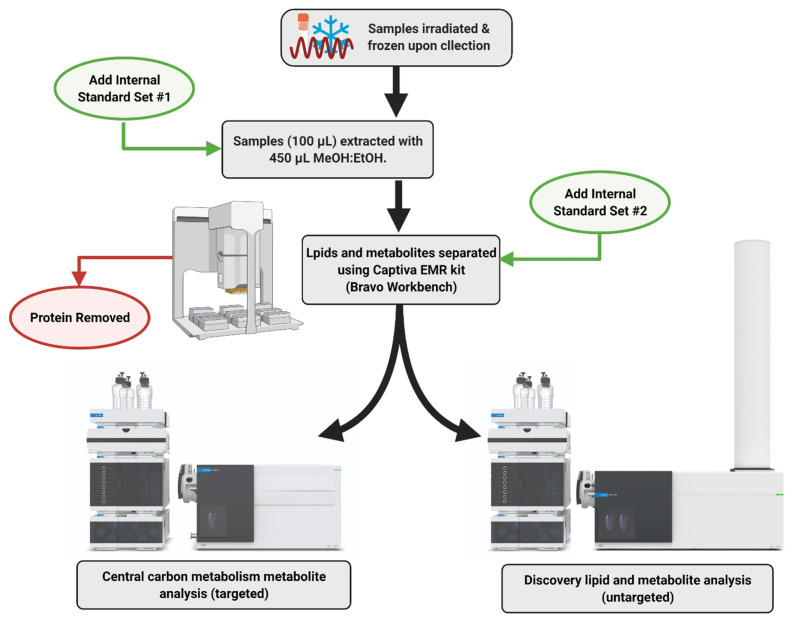
General overview of the sample extraction and analytical workflow of non-invasive samples collected from SARS-CoV-2 challenged ferrets.

A general overview of the sample extraction and analytical workflow is presented in Figure 11. Briefly, thawed 100 µL aliquot of nasal wash, oral swab in PBS, and rectal swab in PBS was added to prefilled tubes containing 450 µL of ice-cold MeOH:EtOH (1:1, *v*/*v*) Samples were then sonicated in an ice bath for 15 min before extraction using the Agilent Bravo Metabolomics Workbench (Agilent Technologies, Santa Clara, CA, USA). The metabolite and lipid extracts were separated via the Captiva EMR-Lipid plate (96-well, 1 mL, 40 mg, Agilent, Mulgrave, Australia). A series of blanks and mixed QC standards were prepared in the same way, without biological material. Pooled biological quality control (PBQC) samples were prepared by combining 10 µL aliquots from each biological sample. The metabolite fraction was dried into a 96-well plate and reconstituted in 50 µL Water:MeOH (4:1, *v*/*v*). Lipids were eluted off the Captiva plate into high recovery glass vials with 500 µL of DCM:MeOH (1:2, *v*/*v*). The lipid extracts were then dried in a speedvac, before reconstituting in 50 µL BuOH:MeOH (1:1, *v*/*v*). Internal standard set #1 comprised 100 ppb of L-Phenylalanine (1-^13^C) and L-Glutamine (amide-^15^N); Internal standard set #2 comprised 200 pb of Succinic Acid (1,4-^13^C_2_), Glycine (1-^13^C), L-Aspartic acid (^13^C_4_), and L-Valine (1-^13^C). The residual relative standard deviation (RDS%) of the internal standards were 6.14% (L-Phenylalanine, 1-^13^C), 3.49% (L-Glutamine, amide-^15^N), 2.04% (Succinic Acid, 1,4-^13^C_2_), 4.79% (Glycine, 1-^13^C), 4.41% (L-Aspartic acid, ^13^C_4_), and 7.64% (L-Valine, 1-^13^C).

### 3.4. Central Carbon Metabolism Metabolomics (LC-QqQ-MS)

Central carbon metabolism metabolites were analyzed using an Agilent 6470 Liquid Chromatography Triple Quadrupole Mass Spectrometer (LC-QqQ-MS) coupled with an Agilent Infinity II ultra-high-performance liquid chromatography (UHPLC) system (Agilent Technologies, Santa Clara, CA, USA). The instrument was operated using the Agilent Metabolomics dMRM Database and Method [57]. Collected data were processed using MassHunter Quantitative Analysis (for QQQ) software (Version 10.0, Agilent Technologies, Santa Clara, CA, USA), normalized to ^13^C L-Phenylalanine, and ^13^C Succinic acid (1 mg mL^−1^, Cambridge Isotope Laboratories, Tewksbury, MA, USA) in preparation for downstream analyses.

### 3.5. Untargeted Polar Metabolites (LC–QToF-MS)

Untargeted polar metabolites were analyzed using an Agilent 6546 Liquid Chromatography Time-of-Flight Mass Spectrometer (LC-QToF) with an Agilent Jet Stream source coupled to an Agilent Infinity II UHPLC system (Agilent Technologies, Santa Clara, CA, USA). Chromatographic separation was achieved by injection (3 µL) of sample onto an Agilent InfinityLab Poroshell 120 HILIC-Z Peek lined column (2.1 × 150 mm, 2.7 µm). Each sample was analyzed in positive ionization mode. The mobile phase was (A) 10 mM ammonium formate in water with 0.1% formic acid and (B) 10 mM ammonium formate in acetonitrile/water (90:10, *v*/*v*) with 0.1% formic acid operated for 20 min with a nonlinear gradient starting at 98% B. The column temperature was set at 25 °C. The detector gas temperature was 225 °C with a drying gas rate of 6 L min^−1^. The sheath gas temperature and flow were 225 °C and 10 L min^−1^; the nebulizer pressure was also 40 psi. The acquisition range was 50 to 1600 *m*/*z*, at 3 spectra per second. Reference mass ions were 922.009198 *m*/*z*. Auto MSMS data on polled PBQC samples were obtained at collisions of 10 eV, 20 eV and 40 eV. The PBQC AutoMSMS data was used to generate a curated PCDL for further interrogation of acquired samples using accurate mass, MS2 spectra and retention time. Collected data were processed using MassHunter Profinder software (Version 10.0, Agilent Technologies, Santa Clara, CA, USA), normalized to IS, and putatively identified against the Agilent METLIN (MS/MS) Metabolite PCDL (G6825-90008, Agilent Technologies, Santa Clara, CA, USA) and a curated in-house PCDL based on MSMS spectra and library threshold score of 0.8.

### 3.6. Untargeted Lipidomics (LC–QToF-MS)

Untargeted lipids were analyzed using an Agilent 6546 Liquid Chromatography Time-of-Flight Mass Spectrometer (LC-QToF) with an Agilent Jet Stream source coupled to an Agilent Infinity II UHPLC system (Agilent Technologies, Santa Clara, CA, USA). Chromatographic separation was achieved by injection (1 µL) of the sample onto an Agilent InfinityLab Poroshell HPH-C18 column (2.0 × 150 mm, 2.7 µm). Each sample was analyzed in positive and negative ionization mode. The mobile phase was (A) 10 mM ammonium acetate and 10 µM medronic acid in water/methanol (90:10, *v*/*v*) and (B) 10 mM ammonium acetate in acetonitrile/methanol/isopropanol (20:20:60, *v*/*v*/*v*) operated for 30 min with a nonlinear gradient starting at 55% B. The column temperature was set at 60 °C. The detector gas temperature was 250 °C with a drying gas rate of 11 L min^−1^. The sheath gas temperature and flow were 300 °C and 12 L min^−1^; the nebulizer pressure was also 35 psi. The acquisition range was 50 to 1600 *m*/*z*, at 3 spectra per second. Capillary voltages for the positive and negative ionization modes were 3500 V and 3000 V, respectively. Reference mass ions were 121.060873 *m*/*z* and 922.009198 *m*/*z* (positive mode), and 119.036320 and 980.016375 *m*/*z* (negative mode). Auto MSMS data on polled PBQC samples were obtained at collisions of 20 eV and 35 eV. Collected data were processed using MassHunter Profinder software (Version 10.0, Agilent Technologies, USA), normalized to IS, and putatively identified against the Agilent METLIN Lipids PCDL (G6825-90008, Agilent Technologies, Santa Clara, CA, USA) and a curated in-house PCDL based on MSMS spectra and library threshold score of 0.8.

### 3.7. Statistical Analysis and Data Integration

The metabolomics and lipidomic data were subjected to further statistical analysis using multivariate statistics. The data were first imported, matched by sample identifiers (metadata), and log-transformed to normalize the data using SIMCA 16.02 (MKS Data Analytics Solutions, Uméa, Sweden). Partial Least Square-Discriminant Analysis (PLS-DA) was performed by finding successive orthogonal components from the SARS-CoV-2 isolate and sample type-specific datasets with maximum squared covariance and was subsequently used to identify the common relationships among the multiple datasets. All models were cross validated using CV-ANOVA in SIMCA, which is a diagnostic approach for assessing the reliability of PLS and OPLS models.

MetaboAnalyst 5.0 (Xia Lab, McGill University, Montreal, QC, Canada) was used for the enrichment and metabolic pathway analysis [58] and metabolites with Benjamini–Hochberg adjusted *p*-value of ≤ 0.05 and, Fold Changes (FC) of ≤0.67 (downward regulation) or >1.5 (upward regulation), were considered to be statistically significant [59]. Chemical clusters based on structural similarity were created for metabolic examination using the ChemRICH analysis [30].

## 4. Conclusions

COVID-19 is a contagious respiratory disease that is causing significant morbidity and mortality. Understanding the impact of a SARS-CoV-2 infection on the host metabolism and how the metabolism is altered in response to different SARS-CoV-2 isolates and variants is still in its infancy, but of great importance to better understand the short- and long-term health impacts of COVID-19. Herein, we investigated the metabolic profile of minimally-invasive biological samples collected from SARS-CoV-2 infected ferrets during viral shedding and post-shedding periods. The samples consisted of oral swabs, rectal swabs and nasal washed. Ferrets (n = 6) were challenged with two Australian SARS-CoV-2 isolates, betaCoV/Australia/VIC01 and betaCoV/Australia/SA01 at different challenge doses. Fragments of SARS-CoV-2 RNA were only found in the nasal wash samples in four of the six ferrets, and in the samples collected 3 to 9 days post-infection (dpi). Central carbon metabolism metabolites were analyzed during viral shedding and post-shedding periods using a dMRM database and method. The differentiated central carbon metabolism metabolites were then subsequently used to guide untargeted metabolomics and lipidomics analysis of the same samples using an LC-QToF-MS methodology.

Multivariate analysis of the acquired data identified 29 significant metabolites and three lipids that were subjected to pathway enrichment and impact analysis. The presence of viral shedding coincided with the challenge dose administered and significant changes in the citric acid cycle, purine metabolism, and pentose phosphate pathways, amongst others, in the nasal wash samples. Glutamate metabolism and, arginine and proline metabolism were the most significantly perturbed pathways in oral swab samples. Glycolysis, amino sugar metabolism and gluconeogenesis was significantly altered in rectal swab samples. Interestingly, a comparison of the host response to SA01 and VIC01 isolates showed that most of the abovementioned metabolites (except thiamine metabolism intermediates) which resulted in a higher host response, were considerably elevated in SA01 isolate challenged ferrets. These results support other reported metabolomic-based findings found in clinical observational studies and indicate the utility of ferrets as a suitable animal model for further COVID-19 research for the early diagnosis of asymptomatic and mild clinical COVID-19 infections in addition to assessing the effectiveness of new or repurposed drug therapies. Furthermore, the utility of nasal wash samples lends itself to be more practical when considering the translation of these findings to other animal models and humans where it is not easy (or at times, practical) to get rectal swab samples. This study also included an assessment of two locally acquired SARS-CoV-2 isolates that were administered at different challenge dosages to simulate an asymptomatic/mild infection.

Through the utility of an established asymptomatic/mild infection animal model, we demonstrated the value of metabolomics for informing better clinical diagnosis and facilitating therapeutic development via non-invasive sampling. More work is needed to establish a deeper understanding of COVID-19 infections, however, here we demonstrated the application of metabolomics applied to an animal model that facilitates the transition to a new COVID-normal society, minimizing the impact of asymptomatic and mild clinical infections that have the potential to fuel unchecked community transmission events.

## Data Availability

The data presented in this study are available on request from the corresponding author. The data are not publicly available due to animal ethics and intellectual property restrictions.

## Supplementary Materials

The following are available online at https://www.mdpi.com/article/10.3390/metabo11050327/s1, Figure S1: PCA overview of the central carbon metabolite data analyzed in the nasal wash, oral swab, and rectal swab samples pre-infection, during viral shedding, and post-viral shedding. Table S1: Identified significant metabolite clusters during viral shedding and post-shedding using the central carbon metabolism metabolite dataset. Figure S2: Enrichment analysis of the identified metabolites of importance from nasal wash samples. Figure S3: Boxplot representing the data of individual significant metabolites from the key metabolism changes in a SARS-CoV-2 ferret model during viral shedding and post-shedding events. Table S2: Significant metabolites identified from central carbon metabolism metabolite dataset. Table S3: Significant metabolites identified from untargeted metabolomics using LC-QToF-MS. Table S4: Significant lipids identified from untargeted lipidomics using LC-QToF-MS. Figure S4: Boxplot representing the data of individual significant metabolites from the key metabolism changes in a SARS-CoV-2 ferret model infected with the SA01 isolate and VIC01 isolate during virus shedding. Table S5: Mutations (>20% Frequency) in VIC01 and SA01 isolates relative to Wuhan-Hu-1 (NC_045512).
