# Supplementary material for: Metabolic Profiling from an Asymptomatic Ferret Model of SARS-CoV-2 Infection
Source: Metabolites. 2021 May 19;11(5):327. doi: 10.3390/metabo11050327 (PMC8160988; doi:10.3390/metabo11050327)
Supplement: Supplementary file 1 [file metabolites-11-00327-s001.zip › metabolites-1183485-supplementary.pdf]

Supplementary Materials

Metabolic Profiling from an Asymptomatic Ferret Model of SARS-CoV-2 Infection

David J. Beale [1,*], Rohan Shah [1,2], Avinash V. Karpe [1], Katie E. Hillyer [1], Alexander J. McAuley [3], Gough G. Au [3], Glenn A. Marsh [3] and Seshadri S. Vasan [3,4]

[1] Land & Water, Commonwealth Scientific and Industrial Research Organisation, Dutton Park 4102, QLD, Australia; r.shah@csiro.au (R.S.); avinash.karpe@csiro.au (A.V.K.); katie.hillyer@csiro.au (K.E.H.)
[2] Department of Chemistry and Biotechnology, Faculty of Science, Engineering and Technology, Swinburne University of Technology, Hawthorn 3122, VIC, Australia; rshah@swin.edu.au (R.S.).
[3] Australian Centre for Disease Preparedness (ACDP), Commonwealth Scientific and Industrial Research Organisation, Geelong 3220, VIC, Australia; alex.mcauley@csiro.au (A.J.M.); gough.au@csiro.au (G.G.A.); glenn.marsh@csiro.au (G.A.M.); vasan.vasan@csiro.au (S.S.V.)
[4] Department of Health Sciences, University of York, York YO10 5DD, UK
* Correspondence: david.beale@csiro.au; Tel.: +61-7-3833-5774

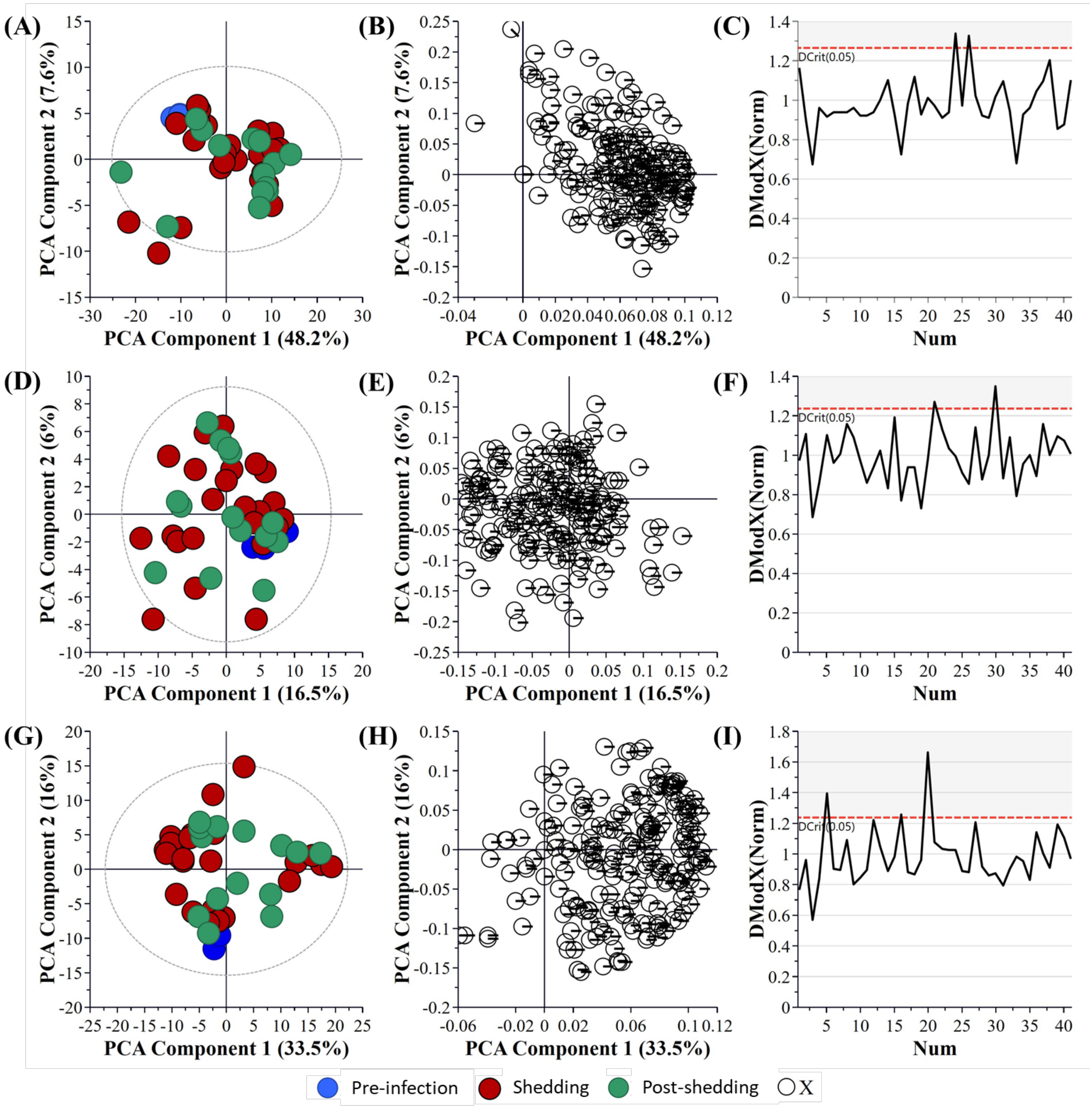

Supplementary Figure S1. PCA overview of the central carbon metabolite data analyzed in the nasal wash (A-C, R_2X = 0.656, and Q_2 = 0.476, DCrit value = 1.2648), oral swab (D-F, R_2X = 0.276, and Q_2 = 0.092, DCrit value = 1.2365), and rectal swab (G-I, R_2X = 0.595, and Q_2 = 0.459, DCrit value = 1.2365) samples pre-infection, during viral shedding, and post-viral shedding. (A, D, G) PLS-DA Score Scatter plot of metabolite data. (B, E, H) PLS-DA Loading Scatter plot of metabolite data. (C, F, I) DModX plot of metabolite data. The ellipse on the loadings scatter plots represents the 95% Hoteling's threshold. *Note, the ellipse presented in Figures S2A, S2D, and S2G represents Hotelling's T2 confidence limit (95%). Note: The colored circles in panel "A", "D", and "G" represent each analyzed sample, with the white circles representing the distribution of metabolite features between these groups.*

Supplementary Table S1. Identified significant metabolite clusters (p ≤ 0.1) during viral shedding and post-shedding using the central carbon metabolism metabolite dataset.

Sample Type	Cluster name	Cluster size	P-value	FDR	Key compound	HMDB	KEGG ID	Altered metabolites
Nasal Wash	Indoles	4	0.0075	0.00005	Indoline-2-carboxylate	HMDB0002285	NA	1
	Adenine Nucleotides	5	0.0346	0.0066	Adenosine 5-monophosphate	HMDB0000045	C00020	1
	Succinates	5	0.0681	0.0066	Succinic semialdehyde	HMDB0001259	C00232	2
	Deoxycytosine Nucleotides	3	0.0939	0.007	2-Deoxycytidine 5-diphosphate	HMDB0001245	C00705	2
	Dicarboxylic Acids	5	0.0190	0.0095	Citramalic acid	HMDB0000426	C00815	0
	Glutarates		0.0848	0.013	3-Methylglutaric acid	HMDB0000752	NA	0
	Guanine Nucleotides	6	0.0647	0.024	Deoxyguanosine 5-triphosphate	HMDB0001440	C00286	3
	Hydroxy Acids	4	0.0190	0.024	Mevalonic acid	HMDB0000227	C00418	1
	Hydroxybenzoates	6	0.0886	0.028	m-Hydroxybenzoic acid	HMDB0002466	C00587	3
	Pentoses	3	0.0086	0.061	L-Arabinose	HMDB0000646	C11476	2
	Pentanols	2	0.0716	0.061	Isopentyl acetate	HMDB0031528	C12296	1
	Pentose phosphates	5	0.0650	0.061	2-Deoxyribose 5-phosphate	HMDB0001031	C00673	1
	Sialic Acids	1	0.0778	0.065	N-Acetylneuraminic acid	HMDB0000230	C19910	1
	Tricarboxylic Acids	5	0.0157	0.12	trans-Aconitic acid	HMDB0000958	C02341	2
Oral Swab	Succinates	5	0.039	1	Argininosuccinic acid	HMDB0000052	C03406	2
Rectal Swab	Hexose phosphates	4	0.0076	0.33	D-Galactosamine	NA	C02262	2
	Hydroxy acids	4	0.027	0.59	Lactic acid	HMDB0144295	C01432	2

FDR: False Discovery Rate; HMDB: Human Metabolome Database; KEGG: Kyoto Encyclopedia of Genes and Genomes

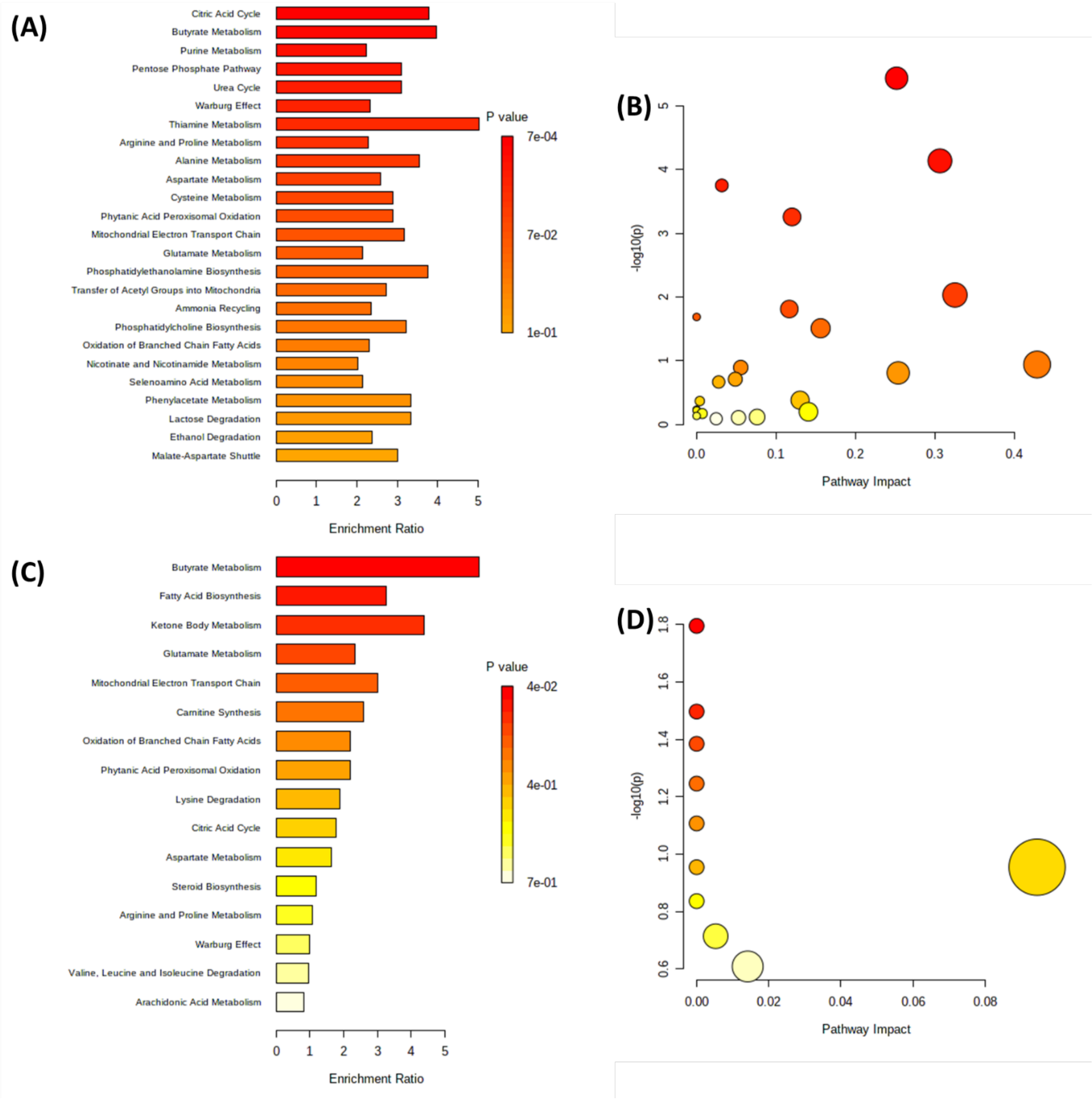

Supplementary Figure S2. Enrichment analysis of the identified metabolites of importance from nasal wash samples. (A) Metabolite enrichment analysis. (B) Metabolite pathway impact analysis. (C) Lipid enrichment analysis. (D) Lipid pathway impact analysis.

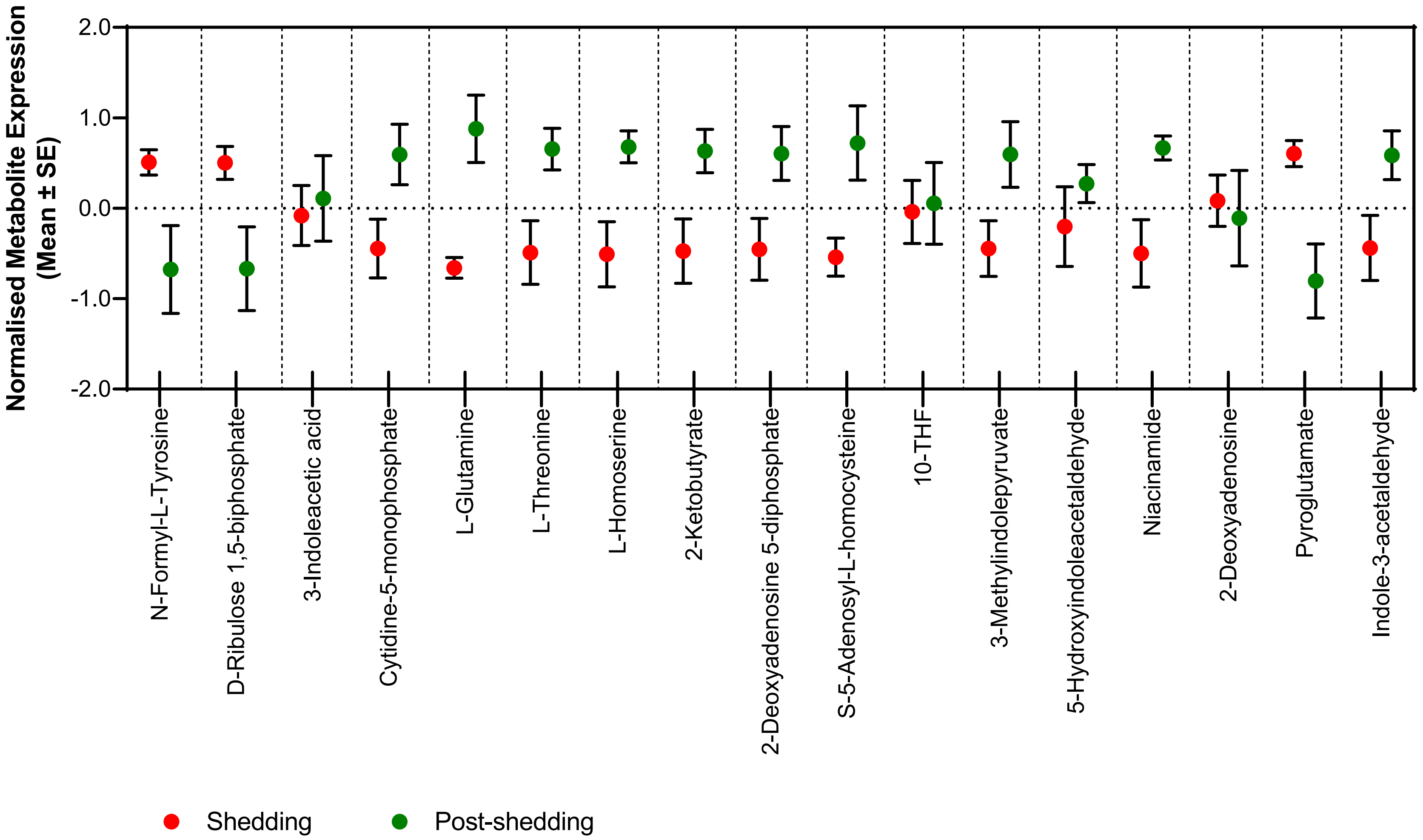

Supplementary Figure S3: Boxplot representing the data of individual significant metabolites from the key metabolism changes in a SARS-CoV-2 ferret model during viral shedding and post-shedding events. Normalized metabolite expression is determined as the mean ± the standard error.

Supplementary Table S2. Significant metabolites identified from central carbon metabolism metabolite dataset.

Metabolite	log2 (FC)	-log10 (p-value)	KEGG ID	HMDB ID	Elevated in
L-Cystine	2.7682	2.1375	C01420	HMDB0000192	SA01
L-Methionine	2.5291	1.5963	C01733	HMDB0033951	
L-Histidine	2.0048	3.2117	C00135	HMDB0000177	
Pyridoxal hydrochloride	1.8407	2.4058	C00250	HMDB0001545	
Cytidine 5-diphosphate	1.1293	1.481	C00112	HMDB0001546	
L-Glutamine	0.76587	9.3871	C00064	HMDB0000641	
L-Threonine	0.74125	3.3936	C00188	HMDB0000167	
L-Homoserine	0.64845	2.9521	C00263	HMDB0000719	
L-Dihydroorotic acid	0.62133	2.0999	C00337	HMDB0003349	
2-Deoxyribose 5-phosphate	0.58836	5.2058	C00673	HMDB0001031	
Itaconic acid	-0.62578	2.5257	C00490	HMDB0002092	VIC01
D-Gluconic acid	-0.69118	1.9507	C00257	HMDB0000625	
N-Formyl-L-Tyrosine	-0.77537	2.0569	No result	No result	
Shikimic acid	-0.78933	2.5124	C00493	HMDB0003070	
N-Acetyl-alpha-D-glucosamine 1-phosphate	-0.8396	3.2117	C04256	HMDB0001367	
N-Acetyl-D-glucosamine 6-phosphate	-0.8396	3.2117	C00357	HMDB0001062	
N-Acetyl D-galactosamine	-0.8726	1.5963	C01132	HMDB0000853	
Ketovaleric acid	-0.94453	2.477	C06255	HMDB0001865	
L-Malic acid	-1.0265	2.1663	C00711	HMDB0000744	
3-Methylglutaric acid	-1.0438	3.2427	No result	HMDB0000752	
Cellobiose	-1.053	1.3492	C06422	HMDB0000055	
4-Guanidobutyric acid	-1.0534	2.1375	C01035	HMDB0003464	
L-Hydroxyglutaric acid	-1.1278	1.912	C02630	HMDB0059655	
Mevalonic acid	-1.1278	1.912	C00418	HMDB0000227	
Dihydroxyacetone phosphate	-1.2088	1.5963	C00111	HMDB0001473	
2-3-Dihydroxyisovalerate	-1.3219	1.3492	C04039	No result	

Supplementary Table S2 (continued). Significant metabolites identified from central carbon metabolism metabolite dataset.

Metabolite	log2 (FC)	-log10 (p-value)	KEGG ID	HMDB ID	Elevated in
alpha-D-Glucose-1-phosphate	-1.4665	1.3535	C00103	HMDB0001586	VIC01
Oxamic acid	-1.4678	5.0099	C01444	No result	
Homocitrate	-1.4685	1.3492	C01251	HMDB0003518	
D-Fructose 6-phosphate	-1.6229	1.5051	C00085	HMDB0000124	
2-Deoxyinosine	-1.6479	1.4177	C05512	HMDB0000071	
L-Glutamic acid	-1.7373	2.1375	C00025	HMDB0000148	
4-Methyl-2-oxovaleric acid	-1.8268	2.6782	C00233	HMDB0000695	
2-3-Dihydroxybenzoic acid	-1.8476	2.6638	C00196	HMDB0000397	
Uridine 5-monophosphate	-1.9414	2.4376	C00105	HMDB0000288	
D-Ribulose 1,5-biphosphate	-1.9674	2.4058	C01182	No result	
beta-Nicotinamide mononucleotide	-1.9977	1.6314	C00455	HMDB0000229	
5-Hydroxy-3-indoleacetic acid	-2.0265	2.1426	C05635	HMDB0000763	
Salicylic acid	-2.0703	2.4058	C00805	HMDB0001895	
2-Deoxyadenosine	-2.1486	1.8156	C00559	HMDB0000101	
Orotic acid	-2.3856	2.5257	C00295	HMDB0000226	
Glyceric acid	-2.4357	6.9069	C00258	HMDB0000139	
4-Hydroxybenzoic acid	-2.4671	2.5257	C00156	HMDB0000500	
Xanthosine	-2.5255	2.5257	C01762	HMDB0000299	
m-Hydroxybenzoic acid	-2.8056	2.5257	C00587	HMDB0002466	
DL-2-Aminoadipic acid	-3.1699	3.2117	C00956	HMDB0000510	
3-Hydroxyphenylacetic acid	-3.1823	2.8092	C05593	HMDB0000440	
Arabinose-5-phosphate	-3.3687	4.5317	C01112	HMDB0011734	

Supplementary Table S3. Significant metabolites identified from untargeted metabolomics using LC-QToF-MS.

Putatively Identified Metabolites	Mass Error (ppm)	log2 (FC)	-log10 (p-value)	KEGG ID	HMDB ID	Elevated in
L-Serine	-7.61	4.1948	4.3568	C00065	HMDB0000187	SA01
L-Leucine	1.52	3.8207	1.3328	C00123	HMDB0000687	
L-Isoleucine	1.52	3.8207	1.3328	C00407	HMDB0000172	
Arachidonic Acid	-10.83	3.8104	1.4151	#N/A	#N/A	
L-Alanine	4.48	3.7569	1.976	C00041	HMDB0000161	
leukotriene-C4	-3.19	3.1122	2.09	C02166	#N/A	
N10-Formyltetrahydrofolic acid	-7.39	3.0032	3.2109	#N/A	HMDB0000972	
4-Hydroxybutanoic acid	6.72	2.0549	2.6513	C00989	#N/A	
4-Hydroxyphenylpyruvic acid	-0.55	1.963	2.6513	C01179	#N/A	
L-Dopa	-5.07	1.743	1.6468	C00355	HMDB0000181	
Thiophene	3.56	1.4386	1.6468	#N/A	HMDB0029718	
5,10-Methylenetetrahydrofolate	-0.87	1.3491	1.7646	C00143	#N/A	
L-Formylkynurenine	16.90	1.2267	2.7231	C02700	HMDB0060485	
L-Tryptophan	3.43	1.2154	1.361	C00078	HMDB0000929	
Nicotinamide adenine dinucleotide	1.06	1.1301	1.3328	C00003	#N/A	
D-Glutamine	5.47	0.68765	2.6513	C00819	HMDB0003423	
L-threo-7,8-Dihydrobiopterin	0.418	0.66428	1.3666	C20263	#N/A	
2,5-Dihydroxypyridine	0.90	-0.6266	2.6513	C01059	#N/A	VIC01
N2-Acetyl-L-ornithine	2.30	-1.2455	2.6513	C00437	#N/A	
Niacinamide	1.53	-1.5095	1.3666	C00153	HMDB0001406	
Nicotine imine	7.61	-1.5764	1.7116	#N/A	HMDB0001010	
2-Hydroxyphenylacetic acid	-0.29	-1.6213	1.4151	C05852	HMDB0000669	
2,3,6-Trihydroxypyridine	2.36	-1.6277	2.3912	C03458	#N/A	
Quinic acid	-0.52	-2.0971	2.0255	C00296	#N/A	
2-Oxoarginine	-24.51	-2.2389	1.7726	C03771	HMDB0004225	
Tryptamine	1.57	-2.2553	1.5488	C00398	HMDB0000303	
2-amino-tetradecanoic acid	4.40	-2.4455	3.4059	#N/A	#N/A	
enalaprilat (anhydrous)	0.51	-2.5619	1.6438	C11720	HMDB0041886	
trans-3-Hydroxycotinine glucuronide	2.81	-2.642	1.5187	#N/A	HMDB0001204	

Supplementary Table S3 (continued). Significant metabolites identified from untargeted metabolomics using LC-QToF-MS.

Putatively Identified Metabolites	Mass Error (ppm)	log2 (FC)	-log10 (p-value)	KEGG ID	HMDB ID	Elevated in
2-amino tridecanoic acid	3.58	-2.7872	2.9742	#N/A	#N/A	VIC01
(Z)-5-Oxohex-2-enedioate	0.00	-3.3279	2.0398	C03453	#N/A	
2'-Deoxyuridine	2.63	-3.3498	2.0204	C00526	#N/A	
Maleamic acid	-0.87	-3.4589	3.1193	C01596	#N/A	
5-Hydroxy-L-tryptophan	0.49	-3.4709	3.1564	C00643	HMDB0000472	
N-Acetyl-L-glutamic acid	1.59	-3.5539	4.9153	C00624	#N/A	
5'-Butyrylphosphoinosine	-2.87	-3.6568	4.2144	C06435	#N/A	
2-Keto-6-acetamidocaproate	2.36	-3.9522	3.2715	C05548	HMDB0012150	
5-Hydroxyindoleacetylglycine	-0.03	-4.0211	1.9645	C05832	HMDB0004185	
4-(2-Aminophenyl)-2,4-dioxobutanoic acid	-0.97	-4.0493	3.2687	C01252	#N/A	
Thymidine	0.94	-4.3172	1.4488	C00214	HMDB0000273	
L-Erythrulose	11.17	-6.2344	2.1279	C02045	HMDB0006293	

Supplementary Table S4. Significant lipids identified from untargeted lipidomics using LC-QToF-MS

Putatively Identified Lipids	Mass Error (ppm)	log2 (FC)	-log10 (p-value)	Elevated in
PE(13:0/18:3(6Z,9Z,12Z))	-5.23	1.9716	3.3921	
PE(14:0/22:2(13Z,16Z))	-0.28	0.92763	1.4637	
PE(15:1(9Z)/22:4(7Z,10Z,13Z,16Z))	-2.41	0.92124	1.35	SA01
PE(12:0/18:2(9Z,12Z))	-4.11	0.90112	1.35	
PE(12:0/17:0)	-6.41	0.76407	1.35	
PE(16:1(9Z)/22:6(4Z,7Z,10Z,13Z,16Z,19Z))	1.63	-1.1496	1.35	
PE(12:0/20:4(5Z,8Z,11Z,14Z))	-1.33	-1.2432	1.35	
PE(18:4(6Z,9Z,12Z,15Z)/22:6(4Z,7Z,10Z,13Z,16Z,19Z))	-0.65	-1.3902	1.4541	VIC01
PE(14:0/20:5(5Z,8Z,11Z,14Z,17Z))	1.18	-1.4124	1.4183	
PE(15:1(9Z)/22:6(4Z,7Z,10Z,13Z,16Z,19Z))	1.19	-2.0691	1.7454	

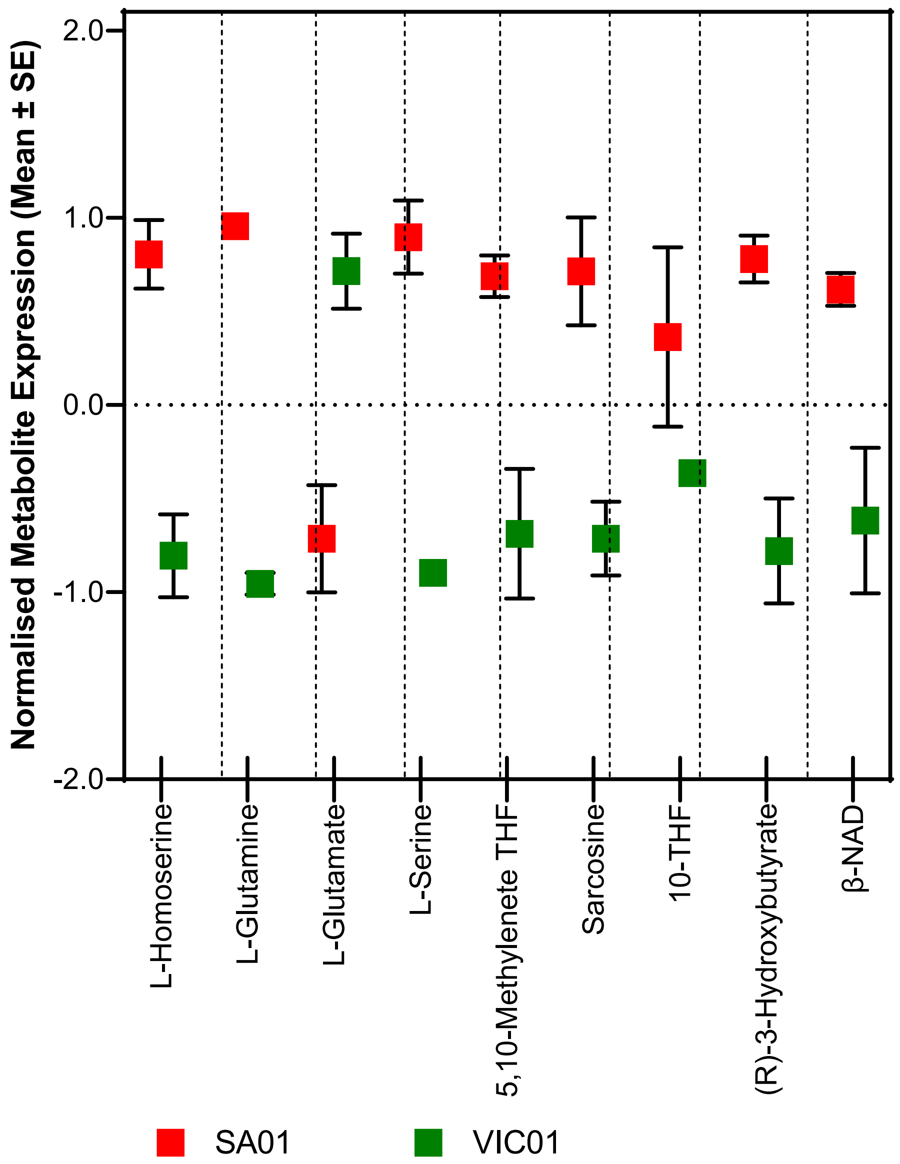

Supplementary Figure S4 Boxplot representing the data of individual significant metabolites from the key metabolism changes in a SARS-CoV-2 ferret model infected with the SA01 isolate and VIC01 isolate during virus shedding. Normalized metabolite expression is determined as the mean ± the standard error.

Supplementary Table S5: Mutations (>20% Frequency) in VIC01 and SA01 isolates relative to Wuhan-Hu-1 (NC_045512)

**Isolate**	**Mutation**	**Effect**	**Frequency (%)**
VIC01	T19065C	Silent	99.81
	T22303G	S247R in Spike	99.94
	G26144T	G251V in ORF3a	99.83
	29750Del (10nt)	10nt Deletion in 3′ UTR	76.51
SA01	C3037T	Silent	99.41
	C17074T	L5604F in orf1ab	99.94
	26284Del	Loss of V14 in Envelope	87.16
	C27213T	Silent	97.88
	T27384C	Silent	99.82
